# Statistical Reports of the Sickness, Mortality, and Invaliding among the Troops

**Published:** 1844-04

**Authors:** 


### Art. II.

1. *Statistical Reports of the Sickness, Mortality, and Invaliding among the Troops.* Compiled by Major Tulloch, *and presented to Parliament,* 1838-41.
2. *Statistical Reports on the Health of the Navy from* 1830 *to* 1836. Compiled by Dr. Wilson, *and ordered by the House of Commons to be printed,* 1840-1.

On various occasions we have brought under the notice of our readers the valuable statistical reports on the health of the army and navy, and have reviewed in detail the leading facts which they tend to establish. We now propose to institute a comparison between the sickness and mortality in these two branches of the public service, and to investigate the causes to which any remarkable difference in this respect is likely to have been attributable.

As a preliminary to this comparison, however, it is necessary to examine those peculiarities in the condition of the soldier and the sailor, by which their health is likely to be affected. Of these the first, and perhaps the most important, is the duration of service. The soldier must enlist for life, or until he is by age or disease unfit for military duty, while the sailor engages only for a limited period, and as ships are generally paid off after being about four years in commission, he is then at liberty to terminate his engagement.* The system of unlimited service exerts, we have no doubt, an injurious influence on the health of the soldier, by depriving him of the hope of returning, at a definite period, to his native land, a hope which would probably have the effect of preventing many of those reckless excesses into which he is occasionally led, and would remove that nostalgic depression, which, in the hour of sickness, frequently renders the best efforts of the medical officer unavailing. It follows as a consequence of the limited duration of the sailor's service, that he is more subject than the soldier to frequent medical examinations, at which unhealthy and ineligible men are rejected. When a ship is about to be paid off, the sailors are allowed to volunteer into another without a certificate from the surgeon being required, but they very often, particularly after a long cruise, prefer having their "lark" on shore, and when their money is spent, if they again wish to enter, they must, in the first place, be examined by him and reported fit for service. Thus an opportunity is afforded of getting rid of many men who have become, or are becoming, inefficient, and though all are not necessarily subject to this re-examination, there is reason to believe a very large proportion undergo it. The advantages arising from a selection of lives have been fully ascertained by the experience of assurance offices, and although the same strictness cannot probably be exercised in the selection of seamen, the influence of these repeated examinations must be considerable. In respect of diet, also, the navy has many advantages over the army. Deputy-inspector-general Marshall says,† "The ration of the seaman is not only ample for three meals a day, but these meals may be varied in no small degree, according to the option of individuals, .....the ration of a soldier (in the colonies) will only furnish him with two comparatively scanty meals daily;" and again, "in this country the dinner (of

* This remark applies to the present period of peace. The case was different formerly.
† United Service Magazine for April, 1842, p. 538.

the soldier) is commonly excellent in quality and abundant in quantity, but it is unvarying; the same kind of articles cooked in the same manner from the 1st of January to the 31st of December—

> Que le vent souffle au nord, ou qu'il souffle au midi,
> C'est toujours du bouilli mais jamais du rôti.

At the present time the dinner hour usually is about one o'clock, after which no arrangement is commonly made to furnish the men with any article of food till breakfast next morning, being a period of about nineteen hours," a system which cannot fail to be prejudicial to their health. It is justly remarked in the Naval Report that " variety in diet is gratifying to the palate, facilitates assimilation, and thereby promotes health and vigour."

The influence of exercise in the preservation of health is too well known to require comment, and in this respect the sailor again has the advantage over the soldier ; it is true the latter has his drills and parades, but these, like his dinner, present too much sameness; there is not that variety, that calling into play of all the different muscles, which forms a distinguishing feature in the exercise of the sailor. In the article of dress—an important accessory to health, particularly in tropical climates—there is also a marked difference in the two services: the sailor being unencumbered by anything tending to oppress or prevent the free use of his muscles, while the soldier is buttoned up to the throat, which is confined by a stiff leather stock, his head is burdened with a heavy schako, and his well-filled knapsack on his back weighs, with his cross belts and arms, upwards of sixty pounds.

From the nature of the sailor's duties another great difference arises; while the soldier in time of peace usually remains in the same place for one or it may be two or three years, performing the same unvarying routine of parades and duties, and passing his leisure hours in the same dull round of garrison amusements or dissipation, the sailor's life is one continual change of scene, and his duties are of a much more varied nature. He is thus in a measure exempt from that listlessness which, we fear, often leads in the army to intemperance or other equally pernicious vices. Of late a good deal has been done in providing amusements for the soldiers, but we much doubt whether the excellent intentions of the authors of these measures have been sufficiently seconded by the officers of regiments.

No information is given in the Naval Reports as to the average age of the sailors, and we are consequently unable to compare it with that of the army; but from the much greater number of boys employed, from the opportunities the men have of quitting the navy after a few years' service, and which they frequently avail themselves of, and from the high rate of invaliding, there seems good reason to believe that it is considerably under that of soldiers, a circumstance tending very materially to reduce the comparative amount of mortality.

The numerous opportunities enjoyed by the navy of sending home their invalids before disease has made such ravages as to render recovery almost impossible, must likewise assist in diminishing the mortality in that service. Most beneficial effects also arise from the frequent changes between one part of a command and another. On this point Dr. Wilson remarks: " After a certain period of service in the West Indies there is often so much diminution of organic force as to render men of little original power, or of advanced years, incapable of laborious duty, and to make them more susceptible of disease, acute and chronic. In these cases, and after attacks of severe disease, when convalescence is slow and uncertain, a run to Halifax, or the gulf of St. Lawrence, acts like a charm. Health, strength, and with them spirits, are acquired at a rate scarcely to be believed by those who have not witnessed their progress." (Intr. to Part i, p. viii.) The same remark obviously applies to other commands having the same variety of climate, but even where the difference in that respect is not so marked "there is much reason for believing that frequent change of climate, so far at least as temperature is concerned, is favorable rather than injurious to health; at any rate there is ground to conclude that such change, though it may not lessen—may even augment the number of attacks, weakens the fatal force of disease." (Part ii, p. 149.)

These constitute some of the most important differences in favour of the navy. On the other hand sailors are, from the nature of their duties, more liable to accidents; they are generally more crowded between decks than soldiers in their barrack-rooms, nor have they the same means of ventilation, and when windsails are employed to remedy this defect, the men in their vicinity are compelled to sleep in a constant current of cold air. They are also much more exposed to cold and wet; as the crew in ships of war is divided into two watches, on which alternately the working of the ship devolves for four hours at a time, one half must always be on deck, whilst at midnight, or 4 a.m., the men relieving the watch rush from their beds into the open air, often inadequately covered and perspiring profusely, and thus pass instantaneously from a heated to a cold, or at least comparatively cold, atmosphere, where they remain during the next four hours. The soldier on the other hand is only on guard every fourth or fifth night, and then, though subject to the inconvenience of remaining in his clothes and accoutrements for twenty-four hours, is not altogether deprived of rest, and, when obliged to leave his (often overheated) guardroom to take his turn of duty as sentry, he is exposed only for two hours at a time, and at night is generally defended from atmospheric vicissitudes by his great-coat and sentry-box.

There are other differences in the relative duties and conditions of these two classes which might have been noticed, but as they depend chiefly on locality, we shall advert to them in our remarks on the sanatory condition of the different commands.

To compare the relative prevalence of diseases in each service, it became necessary to adopt the same system of classification for both. We have preferred that followed by Major Tulloch in the Military Reports, and have, at a considerable expense of time and labour, arranged the others in the same manner. In this we have experienced much difficulty, owing to the want of a column in the naval appendices showing the cause of death in those who died in hospitals abroad, and it has only been by a careful and repeated perusal of the reports that we have been enabled to accomplish our task. As it was also necessary that precisely the same periods should be included in both, and as the Naval Reports extend only from 1830 to 1836, we have constructed tables of the sickness and mortality among the troops for these seven years only, so that the ratios will be found to differ in some particulars from those stated in the reports which comprise, in most instances, a period of twenty years.

The naval commands which can be fairly compared with the military, are the home station, the Mediterranean and Peninsular command, and the East India command, for although the last includes New Holland, the force employed there is so limited as not materially to affect the results, and the operations of the squadron are chiefly directed to the shores of the bay of Bengal, of the coast of Coromandel, and of the island of Ceylon. In the other naval commands, except the South American, climates of such totally different characters are included together as to prevent any accurate comparisons: thus, Western Africa, the Mauritius, and the Cape of Good Hope, form one command, whilst the West Indies and North America comprise another. In South America there are no British troops employed wherewith to form a comparison.

HOME STATION. The ships constituting the home force have been divided in the Naval Report into two classes: the first, comprising flag and harbour duty ships, surveying vessels, and a few cruisers, is wholly employed on the coasts of Great Britain and Ireland and their respective isles, and its cruising operations extend to the British Channel, the North Sea, St. George's Channel, and the Atlantic Ocean, to the limits of the West Indian, African, and Peninsular commands. The second (designated *various*) includes ships which, "though reckoned on home service, are employed occasionally abroad in various detached duties, ships employed in carrying troops, ships fitting out and paying off, and packets while in England." The sickness and mortality in these, will form a fair subject of comparison with that of the dragoon-guards and dragoons in the United Kingdom. It has been found impossible to arrange the *fatal* diseases in classes, owing to the large proportion of deaths in hospitals and sick quarters, the specific causes of which, as already stated, are not detailed in the Naval Reports; our remarks must, therefore, be chiefly confined to the relative prevalence of disease, as shown in the following tables:

| | NAVAL FORCE. | | | | MILITARY. | |
|---|---|---|---|---|---|---|
| | "*Various.*" | | "*Home.*" | | *Dragoon-guards and Dragoons.* | |
| Ratio per 1000 of strength. | Admitted. | Died. | Admitted. | Died. | Admitted. | Died. |
| Fevers .. ... | 60 | | 51 | | 75 | |
| Eruptive Fevers ... | 2 | | 6 | | 3 | |
| Diseases of Lungs ... | 297 | | 300 | | 148 | |
| „ Liver ... | 10 | | 10 | | 8 | |
| „ Stomach & Bowels | 129 | | 106 | | 94 | |
| Epidemic Cholera ... | 4 | | 2 | | 4 | |
| Diseases of the Brain ... | 16 | | 14 | | 6 | |
| Dropsies ... ... | 1 | | 1 | | 1 | |
| Rheumatic Affections ... | 78 | | 86 | | 50 | |
| Venereal „ ... | 176 | | 133 | | 181 | |
| Abscesses and Ulcers ... | 191 | | 175 | | 133 | |
| Diseases of the Eye ... | 15 | | 12 | | 19 | |
| „ Skin ... | 20 | | 20 | | 29 | |
| Other Diseases ... | 78 | | 67 | | 52 | |
| Total, exclusive of Wounds, &c.* | 1077 | 10·5 | 983 | 8·8 | 803 | 13·7 |
| Ratio per 1000 invalided... | 37·1 | | 38·2 | | 26·3 | |

* The class of wounds and injuries has been omitted, as sailors are, from the nature of their duties, much more liable to them than soldiers.

This table shows the mortality among the military to be rather higher than in the naval force, but perhaps not more so than might be accounted for by the difference in their respective ages, and the excess of invaliding in the latter; the case is otherwise, however, as regards the prevalence of disease which is considerably greater in the navy than the army. This will be found chiefly in diseases of the lungs, of the stomach and bowels, and of the brain, in rheumatic affections, and in abscesses and ulcers. While there is a difference in favour of the navy under the head of fevers, and also, as regards ships employed entirely at home, in venereal affections.

*Fevers.* The greater prevalence of this class among the soldiers might have been anticipated from their being quartered in large towns, where the causes of fever usually abound, and also from their more frequent opportunities of indulging in habits which are so apt to give rise to the ephemeral type of this disease. This view seems corroborated by its excess in the "Various" over the "Home" branch of the naval force, as the former includes all ships fitting out, on board of which must necessarily be received men who have been for some time previous in the low haunts of the seaport towns, pursuing a mode of life of the most irregular description.

*Diseases of the lungs.* The most striking difference between the two services is to be found in this class of diseases, and is principally manifested in catarrhal affections, whereof the ratio per 1000 among the dragoon-guards and dragoons has been 122, while in the "Home" division of the navy it amounts to 254, and in the "Various" to 256. This, no doubt, arises from the circumstances already noticed of the more frequent occurrence of night-duty on shipboard, its longer duration, viz. four hours at a time instead of two, the greater exposure of the sailor to the inclemency of the weather, and the sudden chills to which he is liable when turned out of an overheated berth to go on deck in a state of profuse perspiration. Pneumonia and pleuritis are also more prevalent in the navy than army, being in the "Home" division 35, in the "Various" 30, and among the dragoon-guards and dragoons 16. But it is worthy of remark, that while this difference exists in the inflammatory diseases of the lungs, the relative proportion of phthisis and hæmoptysis is to within a fraction the same. Their distribution, however, differs little in the two services, probably owing to the greater facility of discharging from the navy, whereby patients labouring under hæmoptysis are got rid of, who, if longer retained, would in all probability have become phthisical. The ratio of admissions by these diseases conjointly has been among the military $8\frac{3}{10}$, in the "Home" division $8\frac{1}{10}$, and in the "Various" 8 per thousand. The mortality, however, is not in half so large a proportion in the navy as the army, arising, no doubt, from the opportunities sailors have of quitting the service when they feel themselves unequal to their duty. The eagerness which individuals display to revisit their native place in the hope of regaining health, when, in the early stages of consumption, they feel their strength gradually failing and an unwonted languor affecting them, is well known; and, taken in conjunction with the facility with which sailors can obtain their discharge, may we think quite account for the discrepancy noticed by Dr. Wilson between the cases of phthisis and the resulting mortality, without calling in question the accuracy of diagnosis of the naval medical officers.

*Diseases of the stomach and bowels.* The prevalence of diseases of this class is nearly the same among the military and in the "Home" division of the naval force, but their distribution is somewhat different. Thus diarrhœa and cholera (not epidemic) were more prevalent among the soldiers, while colic, dyspepsia, and constipation, were much more frequent among the sailors. This doubtless has arisen from the nature of their respective diets, the free use of vegetables, on the one hand, with the necessity for subsisting to a considerable extent on salt provisions, on the other, being very likely to produce these opposite effects. The military suffered less than the naval force from inflammatory affections of the bowels, but the total amount of these was so trifling as scarcely to affect the general results. A considerable number of cases of dysentery appear in the returns of the "Various" force, and are understood to have occurred in ships being paid off after their return from tropical stations.

*Diseases of the brain.* In this class the difference is chiefly in the greater number of cases of headach and vertigo, in the navy; epilepsy is also more frequent, but we are unable to state from what cause. The other diseases of this class are much the same in both services. Delirium tremens is of rare occurrence, the ratio among the military amounting to 6 per 10,000 of the strength, in the "Home" division of the navy to 7, and in the "Various" to 10 per 10,000; but the mortality in the last has been lowest, one death only having taken place among about 16,000 men, while in the "Home" it was in the proportion of one in 7000, and among the dragoon-guards and dragoons of one in 11,000 of the mean strength respectively. This very low ratio among men whose habits are reputed, and we fear with too much truth, to be very dissipated, affords an additional testimony to the accuracy of our opinion that too great stress has been laid, particularly by some army-surgeons, on intoxication as the chief cause of the high rate of mortality among soldiers.

The excess of the *rheumatic*, as of the *catarrhal affections*, seems to depend on the difference of the duties and exposure to wet and cold in the two services, while the exemption from venereal probably arises from the sailor having fewer opportunities of contracting it.

The only other class of diseases in which a marked difference exists, is that of *abscesses* and *ulcers*, arising from the larger number of cases of slight superficial inflammations of the extremities generally terminating in abscess. Dr. Wilson attributes them in a great measure to the sailors' duties, especially cleaning the decks by washing and stoning. The inflammation is generally confined to the lower extremities and seldom extends above the knee. It seems also not unlikely that this peculiarity may in part depend on the stimulating qualities of the salt water with which their feet and legs are so constantly wet.

While the admissions into hospital in the army from wounds and injuries are about 10 per cent. lower than in the navy, the mortality by this class is nearly the same in the two services, the relative proportion of deaths to cases being increased by the greater number of suicides among the soldiers, a crime, we are happy to observe, scarcely known in the navy. It is true that some of the deaths entered as accidents in the naval returns may have been cases of self-destruction, by the sailor voluntarily throwing himself overboard, but there seems no reason to doubt that they are of much less frequent occurrence than in the army. Sailors are generally a more cheerful race, and frequent change of scene tends to dissipate ennui.

MEDITERRANEAN COMMAND. In the army this comprises the troops at Gibraltar, Malta, and the Ionian islands; while in the navy the ships employed on the coasts of Spain and Portugal, as well as in the Mediterranean, are included; the principal naval station, however, is Malta. Although the services of the naval force are not wholly confined to the Mediterranean, yet as the greater part is employed there, and as the climate of the Spanish coast is very similar to that of Gibraltar, a fair comparison may be drawn of the relative health of soldiers and sailors when exposed to the same climatorial agencies.

The following table shows the ratio of admissions and deaths among every thousand men, on the average of seven years (1830-36), from the principal classes of diseases, exclusive of those by wounds and injuries.

| | NAVAL FORCE. | | MILITARY. | |
|---|---|---|---|---|
| | *Ratio per* 1000. | | *Ratio per* 1000. | |
| | Admitted. | Died. | Admitted. | Died. |
| Fevers ... ... ... ... | 84 | 1·7 | 211 | 3·7 |
| Eruptive Fevers ... ... ... | 4* | 2 | 1 | ·05 |
| Diseases of Lungs ... ... ... | 251 | 3·3 | 144 | 6·5 |
| ,, Liver ... ... ... | 10 | ·3 | 16 | ·6 |
| ,, Stomach and Bowels ... | 158 | 1·1 | 188 | 2·5 |
| Epidemic Cholera ... ... ... | 2 | ·4 | 7 | 2·1 |
| Diseases of Brain ... ... ... | 18 | ·9 | 11 | 1·1 |
| Dropsies ... ... .. ... | 1 | ·2 | 2 | ·4 |
| Rheumatic Affections ... ... | 65 | 1·1 | 44 | 1·05 |
| Venereal ,, ... ... ... | 100 | | 107 | |
| Abscesses and Ulcers ... ... | 283 | | 122 | |
| Diseases of Eyes ... ... ... | 13 | | 55 | |
| ,, Skin ... ... ... | 17 | | 14 | |
| Other Diseases ... ... ... | 71 | | 58 | |
| Total, exclusive of Wounds and Injuries ... | 1077 | 9·2 | 980 | 18 |
| Ratio per 1000 invalided ... ... | $25\cdot\frac{7}{10}$ | | $9\cdot\frac{5}{10}$ | |

This table shows the amount of sickness in the navy to be one tenth higher than in the army in this command, while the mortality from disease among the soldiers is nearly double that of the sailors.

We shall briefly examine in what classes this remarkable difference is principally to be found.

*Fevers.* A large proportion of the cases being of endemic origin, it is not surprising that the troops should suffer in a much higher ratio than the sailors, for while the former remain for a considerable period in the

* In this calculation we have omitted 203 cases of vaccination, as it can scarcely be deemed a disease. All soldiers who do not bear distinct marks being vaccinated immediately on enlistment, cowpox is almost never met with in the military returns from foreign stations: such, however, does not seem to be the practice in the navy; and it would therefore be unfair to include these cases in a comparison of the diseases affecting the two services.

same place exposed to the cause of the disease, the latter are not only frequently changing their station, but generally lie at some distance from the shore, beyond the influence of noxious miasmata. Moreover, a large proportion of the military are stationed in the Ionian islands, where the causes of intermittent and remittent abound, while comparatively few ships are employed there. The influence of this circumstance may be estimated by the fact, that the ratio of fevers among the troops in these islands, amounting to about 2-5ths of the whole force in the Mediterranean, during the seven years under review has been 334, while among those at Gibraltar and Malta, it has only been 133 per 1000.

The intensity of these diseases has been very similar in both services, one death having occurred in 56 cases among the sailors, and one in 57 among the soldiers.

*Eruptive fevers.* Of this class, smallpox and measles have been more prevalent in the naval than the military force, the others have existed to nearly the same extent in both services. In the period under review, 141 cases of smallpox occurred in the navy and only 12 in the army; of the latter, 10 occurred at Malta in 1830, when the disease was epidemic on the island. It prevailed in the navy at Malta in 1830, and at the Tagus in 1833-4, and 6. These reports bear important testimony to the protective value of vaccination, for while at Malta 1169 of the civil population were cut off by the epidemic of 1830-1, only 10 cases and two deaths occurred among the soldiers, and 38 cases and one death among the sailors. Its modifying influence is also evinced, for only 6 per cent. of the cases proved fatal, being somewhat lower than the results of Dr. Gregory's observations on cases protected by vaccination which were admitted into the Smallpox hospital in London. (Med. Chir. Trans., vols. xxii and xxiv.) The cases of measles were three times as numerous in the navy as in the army, attributable to the larger proportion of boys employed, and to the greater difficulty of preventing its extension on shipboard by an effectual separation of the sick.

*Diseases of the lungs.* The admissions by this class in the Mediterranean have, as on the Home station, been twice as numerous in the naval as the military force, from the greater prevalence of catarrhal affections. This, as we have already remarked, probably arises from the nature of their respective duties. The admissions by other diseases of the lungs have been nearly the same in both services except by consumption, of which the proportion among the sailors has been 50 per 10,000, and among the soldiers 67, a difference for which we are unable to assign any satisfactory reason, except the greater facility in the navy of getting rid of subjects who manifest a tendency to this disease. The results, however, corroborate the remarks in the Military Report, that residence in the Mediterranean does not seem to retard the development of consumption in persons constitutionally predisposed to it, for the proportion attacked of the naval force is quite as high as among the civil population at home, and even higher than among sailors on the Home station.

The proportion of deaths by this class of diseases is only one half as high among the naval as the military force, a result which might naturally be expected, from the greater facilities for invaliding, and the more frequent opportunities of sending men home while the disease is in a condition to be benefited by change of climate. The advantages arising from this change have long been admitted in cases of consumption, and in the Naval Report we find it stated that, "though the fact is opposed to many preconceived opinions, it is ascertained that bronchitis, which originates in the Mediterranean, and which often runs rapidly to a fatal termination there, may be arrested by moving the subject to England." That the results in the navy are materially influenced by the facility of invaliding, is corroborated by the fact, that while one half of the cases of hæmoptysis and phthisis among the soldiers terminated fatally, only one fourth of the sailors attacked by them are reported to have died.

*Diseases of the liver* are much more prevalent and twice as fatal among the soldiers as the sailors, but in neither service are they a source of much inefficiency.

*Diseases of the stomach and bowels.* On the Home station we found this class to be rather more prevalent in the navy than the army, but in the Mediterranean command the reverse is the case, in the proportion of 188 to 158. This excess among the military arises entirely, however, from the greater prevalence of these diseases at Gibraltar for while the ratio in that garrison has, during the seven years under observation, amounted to 238 per 1000, it has throughout the other stations in the command only averaged 160, or almost the same as the navy. This most probably is in consequence of the difference in the diet of the soldier, whose ration at Gibraltar during the period under review, consisted of salt beef or pork four times a week in winter, and twice a week in summer; vegetables also were often too expensive for him to obtain, consequently he had the same disadvantages in this respect as the sailor, without the corresponding variety in the other articles of food. In the Mediterranean the sailor, on the contrary, being much in harbour or along shore, is amply supplied with fresh meat and vegetables.

If we compare the relative prevalence of the particular diseases of this class in the navy and army (exclusive of the troops at Gibraltar, which, for the reasons above stated, may be regarded as an exception,) we find that among the latter dysentery is more than twice as prevalent; that colic, with diarrhœa and cholera, occur in nearly the same proportion in both services, while sailors suffer twice as much from dyspepsia and constipation, and are likewise more subject to inflammation. The mortality by diseases of the stomach and bowels has been scarcely half as high in the naval as in the military force, which might have been anticipated, as there are no diseases in which change of air exerts so favorable an influence as in chronic affections of the bowels. Independent of the frequent removal of the sailor from one part of the command to another, constant opportunities are afforded of sending him to England when deemed necessary, while soldiers are often kept at Malta for some months without the means of transport, however urgent their cases may be. As a consequence of this it may be stated, that 15 soldiers only were discharged for diseases of the liver and bowels out of a strength of 62,300, while 55 sailors were invalided for the same affections, out of a strength of 55,709. Had the former enjoyed the same advantages, there seems good reason to believe the resulting mortality would have been proportionately reduced.

*Epidemic cholera.* This disease was much less prevalent and fatal in the navy than among the troops, although of the latter only those at Gibraltar suffered from it. In 1833 there occurred 57 cases and 17 deaths among the ships in the Tagus, the disease at the same time being very prevalent and fatal in Lisbon. In 1834 it again broke out in the naval force, when 39 cases and 9 deaths took place; of these, 4 cases, two of which proved fatal, were on board the Jaseur, off Gibraltar, the remainder were in the Ringdove and Castor, off Santander where the disease then existed. As soon as the Castor put to sea the cholera ceased on board, and probably to the opportunities of quitting the spot where it originated, may be attributed the exemption enjoyed by the navy from this epidemic, and also the minor intensity with which it prevailed, as similar beneficial results have been found to follow the removal of troops, even to a short distance, whenever that measure has been practicable on shore.

*Diseases of the brain* have been considerably more numerous in the navy, chiefly owing to the large number of cases of headach and vertigo. Exclusive of these the general ratio very nearly corresponds, but the distribution of the several diseases varies in each service; thus in the navy, inflammation of the brain, apoplexy, and epilepsy, have been more prevalent, while in the army the excess has been in cases of paralysis, amentia, mania, and delirium tremens, particularly the last two. There have been 14 cases of coup-de-soleil in the navy, but none among the military. The exemption from mania and delirium tremens probably arises from the sailor having fewer opportunities of habitual indulgence in intoxication, for while his ship is at sea he cannot procure more than the regulated allowance of wine or spirits, and even in harbour can only indulge to excess when he has leave on shore; whereas, the soldier, even when confined to his barrack, has the canteen open to him so long as he has money in his pocket to purchase the means of intoxication.

The mortality by this class of diseases closely approximates in the two services; in the navy more than half of the deaths have been recorded as caused by apoplexy, while very nearly half of those among the military appear under the head of delirium tremens. It seems very extraordinary, that out of 56 cases of amentia and mania among the seamen not one should prove fatal; half of them, however, were invalided.

*Dropsies* are so often the sequel of fevers that their greater prevalence among the military might have been anticipated. Twice as many cases occurred among the troops in the Ionian islands as at the other stations, a result which accords with the higher ratio of fevers there, as already noticed in our remarks on that head.

The nature of the duties being the same here as on the Home station, it was natural to expect a similar excess of *rheumatic affections* among the sailors, provided they were influenced, as we supposed them to be, by this circumstance; and accordingly we find that the ratio has been one half higher than among the military.

There is not the same difference in the prevalence of *venereal diseases* which was found to exist between the military and the "home" division of the naval force, the proportion being nearly the same in both services. This most probably arises from strict regulations being enforced by the police for the exclusion from Gibraltar of all females likely to communicate venereal, and for their detection and subsequent treatment in a Lock hospital in Malta and the Ionian islands. Owing to these precautions this class of diseases is little more than half as prevalent among the soldiers in the Mediterranean as at home.

*Abscesses and ulcers* have been still more numerous among the sailors in this command than on the Home station, depending on the same cause, the prevalence of superficial inflammations of the extremities terminating in small abscesses.

*Erysipelas.* This is included under the head of "Other Diseases." We find it to have been four times as prevalent and fatal in the navy as among the soldiers. Although it does not now rage to the same extent as formerly in the navy, it still appears occasionally as an epidemic in particular ships; for instance, 49 cases occurred on board the Windsor Castle in 1830, and 76 in the Prince Regent in 1831; of the latter, 50 were admitted while the vessel was on the Home station and 26 in the Mediterranean; in neither did the disease prevail to a greater extent the following year than among the rest of the fleet. Sometimes, however, the tendency to erysipelas is so great that it appears after the slightest scratch or contusion, and this predisposition manifests itself on board the same ship for a series of years. The Caledonia (120) afforded a striking illustration of this: in 1831, 18 cases occurred in her while forming part of the home force; in 1832, 5 were admitted previous to and 22 after her arrival in the Mediterranean; in 1833 there were only 3 cases, but in 1834 there were 27; in the following year, 30, and in 1836, 34 cases. The same fact is developed, but not quite so forcibly, in the instances of the Donegal (78) and the Edinburgh (74). The cause of this peculiarity has never been explained, nor can any reason be assigned why this disease should prevail in one ship and not affect another close to her, and apparently in every respect similarly situated. The ratio of erysipelas has been higher in the Mediterranean than in any of the other naval commands.

During the period under review, 172 cases of *palpitation of the heart* have been treated in the navy, while only four appear in the military returns. This might, to a certain extent, be owing to the number of growing lads employed in the former, but the difference appears too great to be thus accounted for, nor can we offer any satisfactory solution. The cases of *carditis* and *pericarditis* have been more numerous in the navy, in the proportion of 14 to 10, which may, we think, be explained by reference to the relative numbers of rheumatic affections. *Hernia* has been three times as common among the sailors as the soldiers, induced most probably by the nature of their duty, and also by the severe falls which they often receive.

We have as yet compared the health of the army and navy in temperate regions only, we shall now consider the relative influence of a tropical climate on them.

The East India command. This naval command extends from the tropic of Cancer to the 45th degree of south latitude, and from the 50th to the 150th of east longitude, the northern limit being the isthmus of Suez, the southern the island of Tasmania. It includes all that part of the coast of Asia bounded by the Indian Ocean, the islands in that ocean, the British possessions of New Holland and Tasmania, and the islands in the North Pacific. The operations of the squadron, however, are chiefly directed to the shores of the bay of Bengal, of the coast of Coromandel, and of the island of Ceylon. Australia has generally one or more vessels on its coasts; occasionally some force has been required before Canton, and cruisers have been employed for the suppression of piracy, principally among the eastern islands. Although, therefore, the service is chiefly intertropical, from the great extent of the command and the variety of the services required, the navy enjoys great advantages in the frequent change from one station to another, with an occasional run to the healthy shores of New Holland. The only portion of the British military force in the East whereof the details have been published, with which the health of this portion of the navy can be compared, is that of the European troops in Ceylon. The following table shows the ratio per 1000 of admissions and deaths, except those caused by wounds and injuries, in each of the forces respectively during a period of seven years.

| | Naval Force. | | European Troops in Ceylon. | |
|---|---|---|---|---|
| | *Ratio per* 1000. | | *Ratio per* 1000. | |
| | Admitted. | Died. | Admitted. | Died. |
| Fevers ... ... ... ... | 178 | 3·1 | 319 | 5·6 |
| Eruptive Fevers ... ... ... | | | | |
| Diseases of the Lungs ... ... | 193 | 1·8 | 79 | 5·6 |
| „ Liver ... ... ... | 29 | 1·5 | 50 | 5· |
| „ Stomach and Bowels ... | 266 | 4·6 | 323 | 19·4 |
| Epidemic Cholera ... ... ... | 17 | 2·4 | 24 | 7·3 |
| Diseases of the Brain ... ... | 41 | ·8 | 7 | 1·4 |
| Dropsies .. ... ... ... | 1 | | 5 | |
| Rheumatic Affections ... ... | 67 | | 37 | |
| Venereal „ ... ... ... | 86 | | 69 | |
| Abscesses and Ulcers ... ... | 274 | ·9 | 166 | 1·9 |
| Diseases of the Eye ... ... ... | 15 | | 75 | |
| „ Skin ... ... | 5 | | 6 | |
| Other Diseases ... ... ... | 53 | | 53 | |
| Total, exclusive of Wounds and Injuries ... | 1225 | 15·1 | 1213 | 46·2 |
| Ratio per 1000 invalided ... ... | 33·6 | | 3·6 | |

The same feature may be observed in this as in the former tables, viz. the greater amount of sickness and the lower rate of mortality in the navy, but it is by no means so marked in regard to the admissions as the deaths. The latter may in some measure be accounted for by the greater influence of age on mortality in tropical than temperate climates, by the higher proportion of invaliding, and by the effect of length of residence on various diseases. If the opinion we have already expressed as to the average age of sailors being considerably under that of the military be correct, there can be no doubt that this must tend very materially to reduce the mortality, as the ratio increases with age much more rapidly in tropical than in temperate climates. For instance, in Ceylon, while the deaths from all causes among soldiers under the age of 25 amounted to 24 per 1000; between 25 and 33, it was 55; between 33 and 40, it was 86; and between 40 and 50 was 127 per

1000 of the strength. Again, the amount of invaliding from the East India command is more than nine times as high in the navy as in the army. This seems to arise, not from any difference in the necessity for invaliding, but from the expense of that remedy operating as a bar to its being more generally adopted in the army. A sailor whose health has become impaired by the climate may be transferred to the first of Her Majesty's ships returning to England and there discharged, unless his health is restored by the voyage at this early stage of his disease; while the soldier is detained till some troop-ship or transport is proceeding home, and as many months may elapse before such an opportunity presents itself, he frequently sinks under the disease in the meantime, or his constitution is so much shattered that he does not live to reach his native land.

We understand that since the publication of these Reports instructions have been issued to the naval commanders on foreign stations to afford every facility for the conveyance home, in ships of war, of invalid soldiers recommended by a medical board for change of climate. This was not the case, however, during the period under review, and the difference in the facility of getting rid of men whose constitutions had suffered from the climate, must have had a material influence on the relative amount of mortality. We shall now briefly notice a few of the more important diseases comprised in the preceding table.

*Fevers.* This class of diseases, as at all the other stations we have compared, was much more prevalent and fatal among the soldiers than among the sailors. It is remarkable, however, that the proportion of deaths to admissions is almost exactly the same in both, being 1 in 59 of the sailors, and 1 in 58 of the soldiers. From this we would infer that the higher ratio among the latter does not arise from the admission of a larger number of slight cases, but from the men being exposed in a greater degree to the exciting causes of fever.

The cases of eruptive fevers have been so few that we have included them in the calculation with other fevers; only 7 cases and 2 deaths occurred in the navy, and 13 cases and 2 deaths among the military, in the course of seven years.

*Diseases of the lungs.* While the actual prevalence of catarrhal affections is considerably less both in the army and navy than either at home or in the Mediterranean, the *relative* proportion in the latter service is increased, being three times as high as among the soldiers. The deaths, however, have been more numerous among the latter, probably from the greater difficulty of sending home invalids. Pneumonia and pleuritis have also been more frequent though rather less fatal among the sailors than among the soldiers. But the exemption enjoyed by them from hæmoptysis and phthisis is even more marked than in the Mediterranean, the proportion attacked being little above one half of what occurs among the military, or if we take phthisis alone it is as 30 to 53. It seems evident then that sailors are considerably less subject to phthisis than soldiers; this may be in part accounted for by the greater prevalence among the latter of fevers, which undoubtedly tend to develop phthisis in persons predisposed to it; while the circumstances already adverted to as likely to maintain the general health of the sailor, such as better diet, exercise, the influence of the sea air, the frequent change from one part of the command to another, may also contribute materially to avert this disease. The proportion of deaths to cases is higher than in the Mediterranean command or on the Home station, probably owing to the greater distance from England, as many of those who die on the passage home might arrive in a fit state to be discharged if the voyage was shorter.

*Diseases of the liver* have been one third more prevalent and three times as fatal in the army as in the navy. We shall immediately advert to the cause of the greater intensity of diseases of this class in the army.

*Diseases of the stomach and bowels.* These have been more prevalent and four times as fatal among the soldiers as among the sailors. Gastritis and enteritis have prevailed to nearly the same extent in both services; dysentery has been three times as common, and the cases of colic a little more numerous than among the sailors, while the ratios of all the other diseases of the bowels have been higher in the navy, but more especially dyspepsia and constipation. In connexion with the subject of dysentery, we find a circumstance noticed in the Military Report which illustrates the importance of frequent and minute inquiries into the condition of the soldiers in our colonies.

"Prior to 1832 the European soldier (in Ceylon) had no bedding, unless a wooden cot or stretcher with a thin coverlid of country cloth termed a cumley could be deemed such; consequently he either lay down at night in clothes which had been saturated with perspiration during the day, or if he undressed, the thin coverlid afforded little or no protection against the rapid diminution of temperature often experienced in that climate during the night. This being supposed in some instances to give rise to dysenteric affections, a mattress filled with the dried husk of the cocoa nut, and blankets have since been issued to him, which have added very materially to his comfort, and it may be presumed also to his health." (Ceylon Report, p. 7.)

The correctness of this inference is supported by the diminished prevalence of diseases of the stomach and bowels since that period, as shown in the following statement:

| | 1830 | 1831 | 1832 | 1833 | 1834 | 1835 | 1836 |
|---|---|---|---|---|---|---|---|
| Ratio of admissions per 1000 by this class of diseases ... ... | 370 | 373 | 361 | 301 | 281 | 242 | 329 |
| | 368 | | | 290 | | | |

Thus it appears that after this change in the condition of the soldier a marked reduction took place, and principally among the cases of dysentery. In 1836 there was an increase in the number of admissions as compared with the three preceding years, which, however, was entirely attributable to the greater prevalence of diarrhœa, and apparently of a mild character; dysentery still continued to decrease, indeed there were fewer cases in that than in any previous year.

The mortality has been higher in the army by all the diseases of this class, but the most striking difference is in that from dysentery, which has been nearly five times as high as in the navy. This may have arisen in some measure from the much larger number of sailors invalided for this cause. In no cases does change of air prove more beneficial than in chronic diseases of the liver and bowels, and we find that while only two soldiers have been discharged for these during the seven years under review, 95 sailors have been invalided out of a smaller force. This must of course materially influence the results, and in addition, the opportunity of sending sailors on an occasional cruise, to the coast of New Holland for instance, when convalescent from dysentery, cannot fail to be productive of the best effects. The shorter duration of the sailor's service, too, tells strongly in his favour, for individuals who have once suffered from dysentery are extremely subject to its recurrence, consequently during the ten years a regiment is stationed in Ceylon, some of the men are repeatedly treated for it, each attack renders them more liable to and less able to resist the succeeding, until at length they sink under it, whereas the sailor being seldom employed more than four years in the command, has a much better chance of returning to a temperate climate before irremediable disease of the bowels sets in.

As in the Mediterranean command, *epidemic cholera* was less prevalent and fatal in the navy, and the proportion of deaths to admissions was only about half what occurred among the military. During the period under review it prevailed in an epidemic form in 1832 among the troops in Ceylon, and cut off a twentieth part of the force. An interesting history of the progress of the epidemic on this and two previous occasions is given by Major Tulloch, but the cause of the disease still remains involved in mystery. Although the military at Trincomalee suffered very severely in 1832, only 9 cases and 3 deaths occurred among Her Majesty's ships in the harbour there; indeed throughout the East India command only 24 cases and 4 deaths occurred in the fleet in that year. The greatest number of cases in the navy in this command occurred in 1833, when 123 were admitted, and 16 died; of these, 98 cases and 8 deaths were on board the Undaunted, which had been a fortnight in the Madras roads refitting when the disease appeared. After raging eleven days, the epidemic ceased, the vessel having in the meantime sailed from Madras; no cause could be assigned for its outbreak on this occasion.

*Diseases of the brain.* The difference in the relative amount of these is much greater than in any of the commands yet investigated, but arises chiefly from the number of cases of headach and vertigo. We do not know to what this is attributable, but it is remarkable that while in the navy 384 cases have been recorded under these heads, only 7 appear to have occurred among the military. Regarding the other diseases of this class, apoplexy and paralysis have been equally prevalent in both services; delirium tremens and mania have been more common in the army, while amentia and epilepsy, but particularly the latter, have prevailed to a considerably greater extent in the navy. The mortality by this class has been greater among the soldiers than among the sailors, but the ratio invalided of the latter has been eight times as high as of the former. It is worthy of remark that in a country where spirits can be purchased at a very low price, and where in addition the men are liable to be exposed to the intense heat of a tropical sun, the proportion of delirium tremens has only amounted to 14 cases in every 10,000 soldiers, and 8 in the same number of sailors.

*Dropsies* have been much more prevalent and fatal among the military. In this class are included 9 cases and 3 deaths of *beri beri*, of which disease none occurred among the sailors.

*Rheumatic affections* are rather less prevalent among the military than in the Mediterranean, but the ratio among the sailors is almost exactly the same as in that command.

The ratio of *venereal affections* is lower among the soldiers than the sailors, the difference being principally in the cases of syphilis. No sanatory regulations are enforced in Ceylon, to which this difference can be attributed.

Although the troops suffer to a great extent from *abscesses* and *ulcers* they will be found to enjoy the same exemption which has already been shown to exist both at home and in the Mediterranean, as compared with the navy. The difference consisted as in the other commands, in the cases of superficial inflammation terminating in abscesses; ulcers were considerably more numerous among the soldiers, being frequently produced by the bite of the hirudo geometra.

*Diseases of the eyes* have been a source of much greater inefficiency in the army than in the navy; this does not appear to have arisen from any sudden outbreak of ophthalmia, but to be a constant result in every year of the period under consideration. This diversity has not been found to prevail on the home station.

There has not been the same difference in the number of cases of *carditis* and *pericarditis* which was observed in the Mediterranean, but 25 admissions from palpitation of the heart are recorded in the naval report, while none appear to have occurred among the military.

With regard to *wounds* and *injuries* we find the same feature to prevail in the Mediterranean and East Indies as at home, that while the inefficiency is much greater in the naval force, the mortality by this class (including accidental and sudden deaths,) is higher among the military. Thus in the Mediterranean the ratio of admissions in the latter was 108 and in the former 123, whereas the deaths were $2\frac{4}{10}$ and $1\frac{8}{10}$ respectively. In the East India command the admissions among the sailors were 195 per 1000, and the deaths $2\frac{2}{10}$, while among the soldiers the cases amounted to 143, and the mortality to $3\frac{6}{10}$ per 1000 of the strength.

We must now conclude our remarks on this very interesting subject, and in doing so may be permitted to express our satisfaction that the volumes under review have not been thrown aside, as a large proportion of the "Blue Books" are, without producing any practical result. In accordance with the suggestions contained in these reports, several measures tending materially to ameliorate the condition of the soldier have been adopted. Of these we may instance the improvement of his rations at Gibraltar and in the West Indies, by the diminution of the amount of salt meat issued; the selection of more healthy sites for barracks in Jamaica, and particularly the removal of great part of the European force to the upland stations of Maroon Town and Newcastle; the rotation system of relief, whereby the period of continuous service in the West Indies has been reduced from ten to three or four years; and, lastly, the recent order authorizing a passage to be granted in ships of war to soldiers invalided on foreign stations. It is gratifying to know that although these measures at first met with considerable opposition, they have proved most successful in their results. There yet remains abundant room for improvement, but we trust the executive will persevere in the good work thus begun, and endeavour by judicious measures to raise to the highest standard the health of our army, bearing in mind the opinion of the greatest military authority of the age, that in military operations "as nothing is so useful as a healthy soldier, and nothing so useless, expensive, and burthensome, as one in hospital, any measure which can be adopted to improve the health, is one of the greatest public utility, and wise economy."*

---

### Art. III.

* Gurwood's Despatches of the Duke of Wellington.

